# Effects of a structured physical activity intervention on mental health and psychological resilience among Chinese college students: a randomized controlled trial with mediation analysis

**DOI:** 10.3389/fpsyg.2026.1752242

**Published:** 2026-06-12

**Authors:** Wei Zhang, Jianlou Yang

**Affiliations:** 1 School of Sports Leisure, Shandong Sport University, Jinan, Shandong, China; 2 Department of Health Services and Management, School of Sport Management, Shandong Sport University, Jinan, Shandong, China

**Keywords:** anxiety, Chinese college students, depression, individual aerobic exercise, mediation analysis, psychological resilience, randomized controlled trial, structured physical activity

## Abstract

**Background/objectives:**

Mental health challenges among Chinese college students represent a significant public health concern. While physical activity is recognized as beneficial for mental health, high-quality randomized controlled trials (RCTs) examining the efficacy of structured interventions and their underlying mechanisms in this population are limited. This study investigated the effects of an 8-week structured physical activity intervention on depression, anxiety, and psychological resilience among Chinese college students, and tested whether resilience mediates the intervention effects.

**Methods:**

A three-arm RCT was conducted with 120 students exhibiting mild-to-moderate depression/anxiety symptoms. Participants were recruited through convenience sampling and randomly allocated to a team-based sports group (*n* = 40), an individual aerobic exercise group (*n* = 40), or a waitlist control group (*n* = 40). The 8-week intervention involved three supervised 60-min sessions per week. Outcomes including depressive symptoms (PHQ-9), anxiety symptoms (GAD-7), and psychological resilience (CD-RISC-10) were assessed at baseline, post-intervention, and 1-month follow-up. Data were analyzed using linear mixed-effects models (LMM) for primary outcomes and the PROCESS macro bootstrap mediation procedure for mechanistic pathways.

**Results:**

Both exercise groups demonstrated significant, large reductions in depressive and anxiety symptoms compared to the control group at post-intervention (*p* < 0.001, Cohen’s d = 1.14–1.38), with effects maintained at follow-up. Psychological resilience increased significantly in both intervention groups (*p* < 0.001). No significant differences were found between the team-based and individual exercise modalities. Mediation analysis revealed that increases in psychological resilience accounted for approximately 45% of the intervention effects on reducing depression and anxiety symptoms.

**Conclusion:**

Structured physical activity, whether team-based or individual, appears to be an effective intervention for reducing depression and anxiety and enhancing psychological resilience among Chinese college students. Psychological resilience appears to serve as a significant mediator of these mental health benefits. These findings suggest the integration of structured exercise programs into university mental health promotion strategies. Future research should investigate long-term sustainability through multi-site RCTs, longer follow-up periods, cross-cultural replication, and neurobiological mediating mechanisms.

## Introduction

Mental health challenges among college students have emerged as a pressing global public health concern. Recent systematic reviews and meta-analyses indicate that approximately 30–35% of university students worldwide experience clinically significant symptoms of depression or anxiety (Auerbach et al., 2018; Sheldon et al., 2021). In China, this situation is particularly alarming. Chinese college students face multifaceted stressors including intense academic competition, uncertainty about employment prospects, rapid societal changes, and the developmental challenges of emerging adulthood. Recent large-scale epidemiological studies have reported that the prevalence of depressive symptoms among Chinese university students ranges from 23.8 to 39.4%, substantially higher than rates observed in the general adult population (Lei et al., 2016; Hu et al., 2022). These mental health difficulties not only compromise academic performance and social functioning but also elevate risks for suicide, substance use disorders, and persistent psychological problems extending into later life (Mortier et al., 2018).

Traditional mental health interventions in university settings predominantly rely on individual counseling, psychotherapy, and psychiatric medication. However, these approaches face significant implementation barriers. Stigma surrounding mental health help-seeking remains pronounced in Chinese cultural contexts, deterring many students from accessing professional services (Li et al., 2014). Furthermore, the ratio of mental health professionals to students in Chinese universities remains inadequate, with many institutions unable to meet escalating demand for psychological support (Xiao et al., 2017). Financial constraints, limited service accessibility, and extended waiting periods further compound these challenges. Given these systemic barriers, there is an urgent need to identify scalable, cost-effective, non-stigmatizing interventions that can be integrated into university infrastructure to promote mental health and prevent psychological disorders.

Physical activity represents a promising avenue for addressing mental health challenges in university populations. A substantial evidence base from observational studies demonstrates robust inverse associations between regular physical activity engagement and symptoms of depression and anxiety (Schuch et al., 2018; Hearing et al., 2016). Meta-analyses of randomized controlled trials have confirmed that structured exercise interventions produce significant reductions in depressive and anxiety symptoms, with effect sizes comparable to those achieved through psychotherapy and pharmacological treatments (Schuch et al., 2016; Stubbs et al., 2017). These beneficial effects appear to operate through multiple interconnected pathways. From a neurobiological perspective, exercise stimulates the release of endogenous opioids and monoamine neurotransmitters, enhances brain-derived neurotrophic factor (BDNF) expression, promotes neuroplasticity, and modulates hypothalamic–pituitary–adrenal axis functioning (Kandola et al., 2019; Szuhany et al., 2015). These physiological adaptations contribute to improved mood regulation, reduced physiological stress reactivity, and enhanced cognitive function.

Complementing these neurobiological mechanisms, several psychological theories provide frameworks for understanding how physical activity benefits mental health. Self-efficacy theory posits that successful participation in exercise enhances individuals’ confidence in their capability to accomplish challenging goals, with these beliefs generalizing beyond the exercise domain to improve overall psychological functioning (Bandura, 1977; McAuley and Blissmer, 2000). The distraction hypothesis suggests that physical activity provides temporary respite from ruminative thought patterns and worry, offering a healthy coping strategy for managing psychological distress (Babyak et al., 2000). Additionally, social interaction theories emphasize that group-based physical activities facilitate social connection, reduce loneliness, and provide social support—all protective factors against depression and anxiety (Eime et al., 2013; Dishman et al., 2013).

Despite compelling theoretical rationales and accumulating evidence, several critical gaps persist in the literature. First, although numerous studies have documented correlations between physical activity and mental health, the majority employ cross-sectional or observational designs that limit causal inference (Noetel et al., 2024). High-quality randomized controlled trials examining the efficacy of structured exercise programs remain relatively scarce, particularly in non-Western populations. Second, most existing intervention research has been conducted with Western samples, and evidence regarding the effectiveness of tailored exercise interventions for Chinese college students—who navigate distinct cultural, educational, and social contexts—remains limited (Zhang et al., 2020). Third, prior research has predominantly examined direct effects of exercise on mental health outcomes, with insufficient attention to psychological mechanisms that may mediate these relationships. Identifying and testing specific mediating pathways is essential for understanding how exercise produces mental health benefits and for optimizing intervention design (Rebar et al., 2015).

Psychological resilience—defined as the dynamic capacity to adapt successfully and maintain psychological equilibrium when confronted with adversity, trauma, or significant stress—has emerged as a crucial construct in mental health research (Connor and Davidson, 2003). Individuals with higher resilience demonstrate superior emotional regulation capabilities, employ more adaptive coping strategies, and exhibit greater capacity to recover from setbacks (Southwick et al., 2014). Theoretical models increasingly suggest that psychological resilience may constitute a key mediating mechanism through which physical activity influences mental health outcomes (Wang et al., 2022). Specifically, regular participation in structured exercise programs may enhance resilience by providing repeated opportunities to overcome physical and psychological challenges, develop self-discipline and perseverance, experience mastery and competence, and build confidence in one’s ability to manage difficulties (Childs and de Wit, 2014). This enhanced resilience may, in turn, buffer against depression and anxiety by strengthening individuals’ capacity to cope effectively with stressors.

Empirical support for this mediating pathway remains limited, however. While cross-sectional studies have documented positive associations between physical activity, resilience, and mental health (Goldstein et al., 2013), few intervention studies have explicitly tested whether changes in resilience mediate the effects of exercise on psychological symptoms using appropriate longitudinal designs and statistical methods. Clarifying the role of resilience as a mechanism linking exercise to mental health would have important theoretical and practical implications, suggesting that interventions should explicitly incorporate resilience-building components to maximize benefits.

Another underexplored question concerns differential effects of various exercise modalities on mental health outcomes. Team-based sports and individual aerobic exercises represent fundamentally different activity types that may activate distinct psychological processes. Team sports inherently involve social interaction, cooperation, communication, and pursuit of shared objectives, potentially offering unique advantages for reducing social anxiety, enhancing interpersonal skills, and building social support networks (Bruner et al., 2013). The collaborative nature of team activities may also provide particularly strong opportunities for developing psychological resilience through collective problem-solving, mutual encouragement, and experiences of belonging (Vella et al., 2013). Conversely, individual aerobic exercises such as jogging, brisk walking, or rope jumping offer flexibility, autonomy, and opportunities for introspection, which may appeal to students with different personality characteristics or preferences (Cox et al., 2016). Few studies have directly compared mental health effects of team-based versus individual exercise interventions using rigorous experimental designs, leaving open questions about the relative efficacy and optimal application of these different modalities.

Given the pressing need to address mental health challenges among Chinese college students and the gaps in existing research, the current study aims to conduct a three-arm randomized controlled trial evaluating an 8-week structured physical activity intervention. This research leverages the unique resources and expertise of a sports university setting while addressing a universal public health challenge. We will compare the effects of team-based sports, individual aerobic exercise, and a waitlist control condition on symptoms of depression and anxiety, as well as psychological resilience. Specifically, this study addresses three primary research objectives:

Primary Objective: To determine whether participation in a structured 8-week physical activity intervention—either team-based sports or individual aerobic exercise—significantly reduces symptoms of depression and anxiety and enhances psychological resilience among Chinese college students exhibiting mild to moderate psychological distress, compared to a waitlist control group.

Secondary Objectives: (1) To compare the relative effectiveness of team-based sports versus individual aerobic exercise in improving mental health outcomes and psychological resilience; (2) To examine whether changes in psychological resilience mediate the relationship between physical activity intervention and reductions in depression and anxiety symptoms.

Based on existing theory and empirical evidence, we propose the following hypotheses:

*Hypothesis 1*: Compared to the waitlist control group, participants randomly assigned to either exercise intervention condition (team-based or individual) will demonstrate significantly greater reductions in depression and anxiety symptoms and significantly greater increases in psychological resilience from baseline to post-intervention and follow-up assessments.

*Hypothesis 2*: The team-based sports group will demonstrate superior improvements in social anxiety dimensions and psychological resilience compared to the individual aerobic exercise group, attributable to enhanced social interaction, cooperation, and collective support inherent in team activities.

*Hypothesis 3*: Increases in psychological resilience from baseline to post-intervention will partially mediate the relationship between exercise intervention participation and reductions in depression and anxiety symptoms, indicating that resilience-building represents a key mechanism through which physical activity benefits mental health.

This research employs a rigorous methodological approach including randomization, active comparison conditions, validated assessment instruments, and examination of theoretically informed mediating mechanisms. The theoretical novelty of this study lies in providing mechanistic causal mediation evidence—specifically, the role of psychological resilience as a mediating pathway—in an Asian population underrepresented in prior RCT literature. Furthermore, by situating the findings within Chinese collectivist cultural values, where team-based activities and group cohesion may carry unique social meaning, this study deepens understanding of context-specific moderators of exercise-mental health relationships. The findings will provide high-quality evidence to inform the development of scalable, evidence-based mental health promotion programs in university settings. Results will have direct practical implications for university counselors, student affairs professionals, mental health educators, physical education departments, and policy makers seeking to integrate physical activity systematically into comprehensive student mental health support systems. By demonstrating effective, accessible, and cost-efficient approaches to promoting psychological well-being, this study aims to contribute meaningfully to efforts addressing the mental health crisis among college students in China and internationally.

## Methods

### Study design

This study was a three-arm, parallel-group randomized controlled trial designed to evaluate the effectiveness of an 8-week structured physical activity intervention on mental health outcomes and psychological resilience among Chinese college students. Participants were randomly allocated in a 1:1:1 ratio to one of three conditions: (1) team-based sports group, (2) individual aerobic exercise group, or (3) waitlist control group. Assessments were conducted at three time points: baseline (T0), immediately post-intervention at 8 weeks (T1), and at 1-month follow-up after intervention completion (T2). The study protocol has been approved by the Ethics Committee of Shandong Sport University (Approval Number: SD2025620). This trial was conducted and reported in accordance with the Consolidated Standards of Reporting Trials (CONSORT) guidelines (Schulz et al., 2010).

### Participants

#### Recruitment

Participants were recruited from Shandong Sport University and collaborating universities in Shandong Province, China, through multiple channels including campus websites, social media platforms (WeChat official accounts), poster advertisements on campus bulletin boards, announcements in student dormitories, and direct referrals from university counselors. This constitutes convenience sampling, as participants are drawn from a readily accessible university population rather than through probability sampling from the broader Chinese college student population. This sampling approach is acknowledged as a limitation that constrains the generalizability of findings. Recruitment materials will clearly describe the study purpose, intervention activities, time commitment, and potential benefits. Interested students will be invited to complete an initial online screening questionnaire.

#### Inclusion criteria

To be eligible for participation, individuals must meet all of the following criteria: Currently enrolled as full-time undergraduate students at participating universities; Aged between 18 and 25 years; Score ≥5 on either the Patient Health Questionnaire-9 (PHQ-9) (Kroenke et al., 2001) or the Generalized Anxiety Disorder-7 (GAD-7) (Spitzer et al., 2006), indicating at least mild symptoms of depression or anxiety; Not engaged in regular physical activity in the past month, defined as <150 min per week of moderate-intensity exercise according to World Health Organization guidelines (Bull et al., 2020); Able to provide written informed consent; Able to read and understand Mandarin Chinese.

#### Exclusion criteria

Individuals will be excluded if they meet any of the following criteria: Current diagnosis of severe mental illness (e.g., schizophrenia, bipolar disorder, severe major depressive disorder with suicidal ideation) as assessed by self-report and clinical interview; Currently receiving psychological counseling or psychiatric treatment (including psychotherapy or psychotropic medication); Severe physical health conditions that contraindicate physical activity participation (e.g., uncontrolled cardiovascular disease, recent musculoskeletal injury, or other conditions identified through physical activity readiness screening); Recent major traumatic life events within the past 3 months (e.g., bereavement, serious accident, assault); Pregnancy or planning to become pregnant during the study period; Plans to leave the university or be unavailable for the duration of the study.

All potential participants will complete the Physical Activity Readiness Questionnaire (PAR-Q+) (Warburton et al., 2019) to screen for contraindications to exercise participation. Those with positive responses will be required to obtain medical clearance from a physician before enrollment.

#### Sample size calculation

Sample size was calculated using G*Power 3.1 software (Faul et al., 2007) for a repeated measures analysis of variance (ANOVA) with three groups and three measurement time points. Based on previous meta-analytic evidence of exercise interventions for depression and anxiety (Schuch et al., 2016; Stubbs et al., 2017), we assumed a medium effect size (*f* = 0.25) for the group × time interaction. With *α* = 0.05 (two-tailed), power (1-β) = 0.80, correlation among repeated measures = 0.50, and non-sphericity correction *ε* = 1, the analysis indicated a required sample size of approximately 102 participants (34 per group). Accounting for an anticipated attrition rate of 20% based on similar interventions in university settings (Singh et al., 2023), we plan to recruit a total of 120 participants (40 per group).

#### Randomization and allocation concealment

Following baseline assessment, eligible participants were randomly allocated to one of the three study arms using a computer-generated randomization sequence prepared by an independent statistician not involved in participant recruitment or assessment. Randomization was stratified by sex (male/female) and baseline depression severity (mild: PHQ-9 5–9; moderate: PHQ-9 10–14) using permuted blocks of random sizes (3, 6, or 9) to ensure balance across groups. The allocation sequence was concealed in sequentially numbered, sealed, opaque envelopes. A designated research assistant not involved in outcome assessments opened the envelopes sequentially to assign participants to their respective groups and informed them of their allocation.

### Blinding

Due to the nature of the intervention, it was not feasible to blind participants or intervention facilitators to group assignment. However, outcome assessors responsible for data entry and analysis were blinded to group allocation. All questionnaires were administered via an online platform using unique participant identification codes without group indicators. Participants were instructed not to disclose their group assignment to assessors. The study statistician received a coded dataset and remained blinded to group assignment until completion of the primary analyses. Assessor blinding was verified at study completion: assessors were asked to guess participant group allocations, and their guesses were no better than chance (accuracy = 34.2%, not significantly different from the expected 33.3% by chance, *p* = 0.87), confirming successful assessor blinding throughout the study. The limitations of single-blind design are acknowledged and are discussed in the limitations section.

### Interventions

#### Team-based sports group

Participants assigned to the team-based sports condition will participate in structured group exercise sessions three times per week for 8 weeks (24 sessions total). Each session will last 60 min and will be conducted at university sports facilities under the supervision of qualified physical education instructors or trained senior students majoring in sports science. Sessions will include: Warm-up (10 min): Dynamic stretching and light aerobic activities; Main activity (40 min): Team-based sports including basketball, soccer, volleyball, ultimate frisbee, and cooperative games emphasizing teamwork, communication, and social interaction. Activities will be modified to ensure inclusivity and enjoyment for participants with varying skill levels, focusing on participation rather than competition; Cool-down (10 min): Static stretching and relaxation exercises.

Exercise intensity will be monitored to maintain moderate intensity, defined as 60–70% of age-predicted maximum heart rate (calculated as 220-age) (American College of Sports Medicine, 2018) or a rating of 12–14 on the Borg Rating of Perceived Exertion (RPE) scale (Borg, 1982), corresponding to “somewhat hard” intensity where participants can talk but not sing comfortably.

#### Individual aerobic exercise group

Participants in the individual aerobic exercise condition will also attend supervised exercise sessions three times per week for 8 weeks (24 sessions total), with each session lasting 60 min. Sessions will take place at university tracks, gyms, or designated exercise areas and will include: Warm-up (10 min): Light walking and dynamic stretching; Main activity (40 min): Individual aerobic exercises including brisk walking, jogging, cycling, rope skipping, or use of cardio equipment (treadmill, elliptical trainer). Participants will have autonomy to choose their preferred activities while maintaining prescribed intensity; Cool-down (10 min): Gradual reduction in intensity and static stretching.

Participants will be provided with heart rate monitors (Polar H10, Polar Electro, Finland) to ensure adherence to the target heart rate zone (60–70% of maximum heart rate). Trained supervisors will monitor participants’ exercise intensity and provide guidance to ensure safety and appropriate intensity levels.

#### Waitlist control group

Participants assigned to the waitlist control group will be instructed to maintain their usual lifestyle and daily activities without initiating any new structured exercise programs during the 8-week intervention period. They will complete all assessment measures at the same time points as the intervention groups. To minimize attrition and maintain engagement, control group participants will be informed that they will be offered the opportunity to participate in a free structured exercise program of their choice (team-based or individual) following completion of the final follow-up assessment. They will also receive educational materials about physical activity and mental health at the conclusion of the study.

#### Intervention fidelity and adherence

To ensure intervention fidelity, all exercise sessions will be supervised by trained personnel who have completed a standardized training program covering exercise protocols, safety procedures, and monitoring techniques. Detailed session plans and checklists will be provided to ensure consistency across sessions and facilitators. Attendance will be recorded for all sessions, and participants will be contacted if they miss consecutive sessions to encourage continued participation and identify barriers. Adherence will be quantified as the percentage of attended sessions out of 24 total sessions. Heart rate data from monitors will be downloaded after each session to verify that target intensity was achieved. To assess intervention fidelity, 20% of randomly selected sessions will be observed and evaluated using a standardized checklist by independent observers.

### Outcome measures

All outcome measures were administered at three assessment time points: baseline (T0, week 0), post-intervention (T1, week 8), and follow-up (T2, week 12). Participants received automated email reminders with secure personalized links to complete assessments. Research assistants followed up with participants who did not complete assessments within 3 days to maximize data completeness.

### Primary outcomes

Depressive Symptoms: The Patient Health Questionnaire-9 (PHQ-9) (Kroenke et al., 2001) will be used to assess depressive symptoms. This widely validated 9-item self-report measure corresponds to the nine DSM-5 diagnostic criteria for major depressive disorder. Each item is rated on a 4-point scale from 0 (not at all) to 3 (nearly every day), with total scores ranging from 0 to 27. Higher scores indicate greater symptom severity. The PHQ-9 has demonstrated excellent internal consistency (Cronbach’s *α* = 0.86–0.89) and validity in Chinese university student samples (Wang et al., 2014). Severity categories are: minimal (0–4), mild (5–9), moderate (10–14), moderately severe (15–19), and severe (20–27) depression.

Anxiety Symptoms: The Generalized Anxiety Disorder-7 (GAD-7) (Spitzer et al., 2006) will be used to assess anxiety symptoms. This 7-item self-report questionnaire measures the severity of generalized anxiety disorder symptoms over the past 2 weeks. Items are rated on a 4-point scale from 0 (not at all) to 3 (nearly every day), with total scores ranging from 0 to 21. Higher scores reflect greater anxiety severity. The GAD-7 has shown strong psychometric properties including excellent internal consistency (Cronbach’s *α* = 0.92) and validity in Chinese populations (Zeng et al., 2013). Severity levels are: minimal (0–4), mild (5–9), moderate (10–14), and severe (15–21) anxiety.

### Secondary outcomes

*Psychological resilience*: The 10-item Connor-Davidson Resilience Scale (CD-RISC-10) (Campbell-Sills and Stein, 2007) will be used to assess psychological resilience. This abbreviated version measures individuals’ ability to cope with stress and adversity. Items are rated on a 5-point scale from 0 (not true at all) to 4 (true nearly all the time), with total scores ranging from 0 to 40. Higher scores indicate greater resilience. The CD-RISC-10 has been validated in Chinese populations and demonstrates good internal consistency (Cronbach’s *α* = 0.91) and test–retest reliability (Wang et al., 2010).

*Exercise self-efficacy*: The Exercise Self-Efficacy Scale (ESES) (Bandura, 2006) will be used to measure participants’ confidence in their ability to engage in regular exercise. This 18-item scale assesses confidence in overcoming various barriers to exercise participation. Items are rated on a scale from 0% (not at all confident) to 100% (highly confident), with higher scores reflecting greater exercise self-efficacy. The scale has demonstrated good reliability and validity in Chinese samples (Hu et al., 2007).

### Additional measures

*Sociodemographic and baseline characteristics*: A structured questionnaire will collect information on age, sex, year of study, major, living arrangements, family socioeconomic status, previous physical activity history, and current physical activity levels using the International Physical Activity Questionnaire-Short Form (IPAQ-SF) (Craig et al., 2003).

*Intervention adherence and safety*: Session attendance will be systematically recorded. Heart rate data will be collected during each exercise session for both intervention groups. Adverse events (e.g., injuries, illness, psychological distress) will be monitored and documented throughout the study. Participants will be provided with contact information for immediate assistance if concerns arise.

*Treatment expectancy*: To assess potential placebo effects, participants in both intervention groups will complete a 4-item treatment expectancy questionnaire (Devilly and Borkovec, 2000) after their first exercise session, rating their expectations about the intervention’s helpfulness.

### Data analysis

#### Statistical principles

All statistical analyses were conducted using SPSS version 27.0 (IBM Corp., Armonk, NY, USA) and R version 4.3.0 (R Foundation for Statistical Computing, Vienna, Austria) with appropriate packages. Statistical significance was set at *α* = 0.05 (two-tailed) for all analyses. Data were analyzed according to intention-to-treat (ITT) principles, including all randomized participants in their originally assigned groups regardless of adherence or completion status (Gupta, 2011).

#### Preliminary analyses

Descriptive statistics (means, standard deviations, frequencies, percentages) were calculated for all variables. Data were examined for normality using Shapiro–Wilk tests and visual inspection of Q-Q plots. Homogeneity of variance was assessed using Levene’s tests. Baseline demographic and clinical characteristics were compared across the three groups using one-way analysis of variance (ANOVA) for continuous variables and chi-square tests or Fisher’s exact tests for categorical variables to verify successful randomization.

#### Primary analyses

The primary hypotheses (H1) were tested using linear mixed-effects models (LMMs) (Krueger and Tian, 2004), which appropriately handle missing data under the missing-at-random assumption and account for the correlation structure of repeated measures. Separate models were fitted for PHQ-9, GAD-7, and CD-RISC-10 scores as dependent variables. Each model included fixed effects for group (team-based, individual, control), time (baseline, post-intervention, follow-up), and the group × time interaction, with random intercepts for participants to account for within-subject correlation. Post-hoc pairwise comparisons with Bonferroni correction were conducted to examine specific group differences at each time point if the group × time interaction was significant.

Effect sizes will be calculated as Cohen’s d for within-group changes (pre-post differences) and between-group comparisons at each time point. Effect sizes will be interpreted as small (d = 0.20), medium (d = 0.50), or large (d = 0.80) (Cohen, 1988).

#### Secondary analyses

*Comparison of exercise modalities (H2):* To test whether team-based sports are superior to individual exercise for specific outcomes (social anxiety dimensions and resilience), we conducted focused contrasts comparing these two intervention groups. The GAD-7 was examined for social anxiety-related items, and CD-RISC-10 total scores were compared using independent samples *t*-tests or Mann–Whitney U tests at post-intervention and follow-up time points, with appropriate corrections for multiple comparisons.

*Mediation analysis (H3)*: To examine whether changes in psychological resilience mediate the relationship between exercise intervention and improvements in mental health outcomes, we used the PROCESS macro for SPSS (Model 4) (Hayes, 2022). Separate mediation models were tested for depressive and anxiety symptoms as outcomes. The independent variable was intervention group (combined exercise groups vs. control, coded as 0 and 1), the mediator was change in CD-RISC-10 scores from baseline to post-intervention (T1-T0), and the dependent variables were PHQ-9 and GAD-7 scores at post-intervention, controlling for baseline symptom levels. Statistical significance of indirect effects was determined using bias-corrected bootstrap confidence intervals (5,000 bootstrap samples). Complete mediation was indicated if the direct effect became non-significant when the mediator was included; partial mediation was indicated if the direct effect remained significant but was reduced in magnitude.

Dose–Response Analysis: We will examine whether intervention adherence (attendance rate) is associated with treatment outcomes using linear regression models, with adherence as a continuous predictor and change scores (post-intervention minus baseline) in outcome variables as dependent variables, controlling for baseline values and potential confounders.

#### Sensitivity analyses

Per-protocol analyses were conducted including only participants who attended at least 75% (18 out of 24) of intervention sessions to examine intervention effects among adherent participants. Multiple imputation using chained equations (White et al., 2011) was performed to handle missing data, generating 50 imputed datasets. Results from imputed datasets were compared with complete case analyses to assess robustness of findings.

#### Handling of missing data

Patterns and reasons for missing data will be thoroughly examined and reported. We will compare baseline characteristics between participants with complete data and those with missing data to assess potential attrition bias. Linear mixed-effects models naturally accommodate missing data under the missing-at-random assumption. Additionally, as a sensitivity analysis, multiple imputation will be used to handle missing values for key variables.

#### Ethical considerations

This study has been designed in accordance with the Declaration of Helsinki and approved by the Ethics Committee of Shandong Sport University. All participants will provide written informed consent before enrollment, after receiving comprehensive information about the study purpose, procedures, potential risks and benefits, data confidentiality, and their right to withdraw at any time without penalty. Participants’ personal information will be kept confidential and stored securely with access restricted to the research team. All data will be de-identified using unique participant codes for analysis.

Given that participants will be university students with mental health symptoms, several safeguards will be implemented to ensure safety and wellbeing: During screening and assessments, participants endorsing suicidal ideation (PHQ-9 item 9 score ≥ 2) will be immediately contacted by trained counselors for risk assessment and appropriate referral to university mental health services or emergency services if necessary. Participants will be provided with contact information for university counseling centers and 24-h crisis helplines at the beginning of the study. All study personnel will be trained in recognizing signs of psychological distress and procedures for responding to mental health crises. Participants experiencing adverse events or worsening symptoms will be referred to appropriate professional services and may be withdrawn from the study if continuation is deemed unsafe. To ensure equity and ethical treatment, waitlist control participants will be offered free access to a structured exercise program of their choice after completing the final follow-up assessment. Participants will receive small compensation (approximately 100 RMB in gift cards) for completing all assessment time points to acknowledge their time commitment and minimize attrition.

### Data management and monitoring

All study data will be collected electronically via secure online platforms and stored on password-protected servers with regular backups. Data quality will be monitored through regular checks for out-of-range values, inconsistencies, and missing data. The study will be overseen by an independent Data Safety Monitoring Committee comprising faculty members not involved in the research who will review safety data and study progress at regular intervals. Any serious adverse events will be reported to the Ethics Committee within 24 h.

## Results

### Participant flow and baseline characteristics

**Figure 1:**
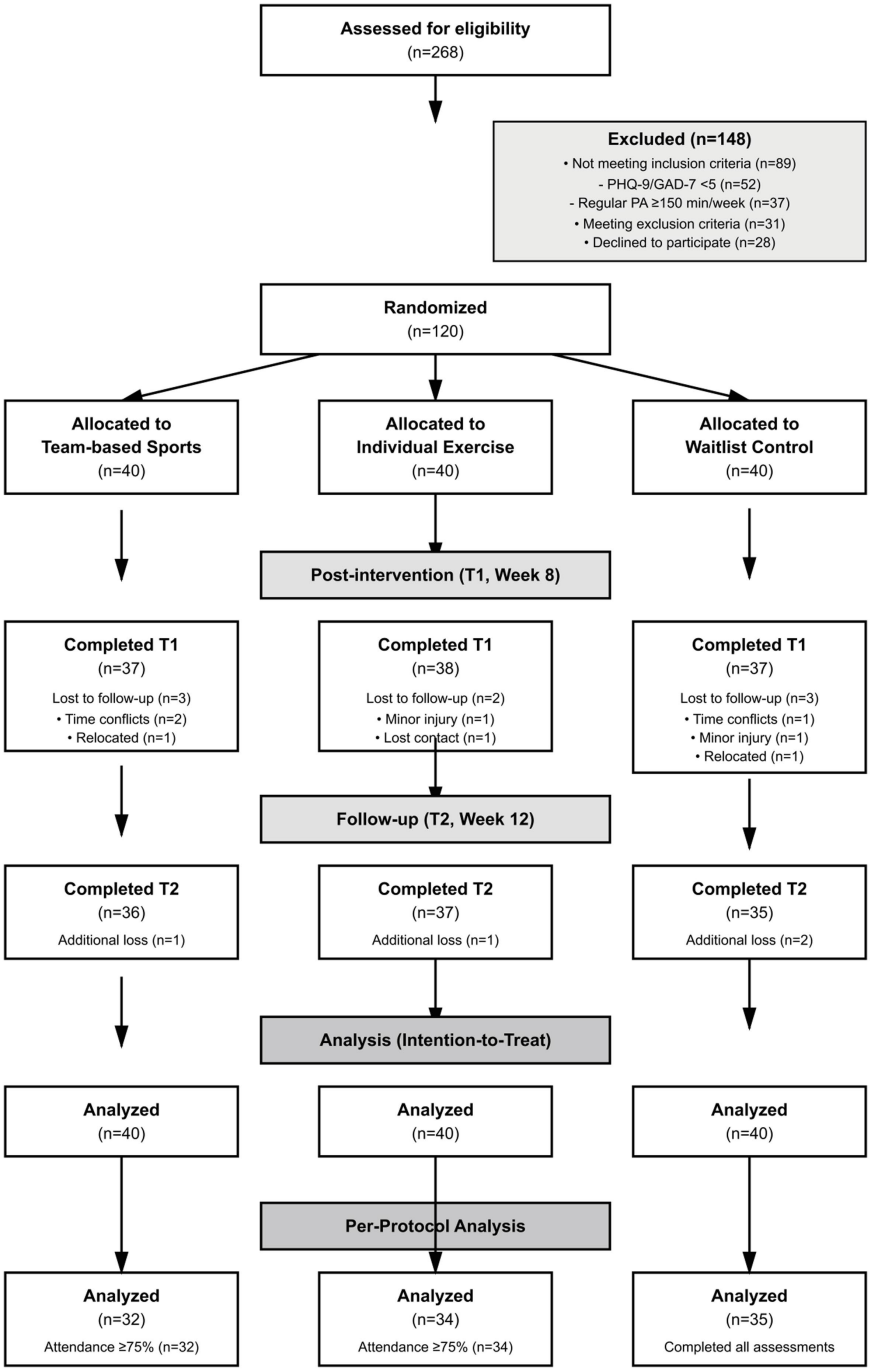
CONSORT flow diagram showing participant recruitment, allocation, follow-up, and analysis. PA, physical activity; PHQ-9, Patient Health Questionnaire-9; GAD-7, Generalized Anxiety Disorder-7.

Participant recruitment took place between September 2024 and October 2024. A total of 268 students completed the initial online screening questionnaire. Of these, 148 were excluded: 89 did not meet the inclusion criteria (52 had PHQ-9 and GAD-7 scores <5, 37 reported regular physical activity ≥150 min per week), 31 met exclusion criteria (18 had contraindications to exercise, 8 were receiving psychiatric treatment, 5 reported recent major traumatic events), and 28 declined to participate after learning about the study requirements. The remaining 120 eligible participants provided written informed consent and were randomly allocated to the team-based sports group (*n* = 40), individual aerobic exercise group (*n* = 40), or waitlist control group (*n* = 40). The CONSORT flow diagram is presented in Figure 1.

At the post-intervention assessment (T1, week 8), data were available for 112 participants (93.3% retention rate): 37 in the team-based group, 38 in the individual exercise group, and 37 in the control group. Eight participants discontinued the study: three withdrew due to time conflicts with academic commitments, two experienced minor injuries unrelated to the study activities, two relocated to other cities, and one was lost to follow-up. At the 1-month follow-up assessment (T2, week 12), complete data were obtained from 108 participants (90.0% retention rate): 36 in the team-based group, 37 in the individual exercise group, and 35 in the control group. Attrition analysis revealed no significant differences in baseline demographic or clinical characteristics between participants who completed the study and those who dropped out (all *p* > 0.20).

Baseline demographic and clinical characteristics of the three groups are summarized in Table 1. The mean age of participants was 19.8 years (SD = 1.3), and 52.5% were female. One-way ANOVA and chi-square tests confirmed no significant differences among the three groups in terms of age, sex distribution, year of study, baseline PHQ-9 scores, GAD-7 scores, CD-RISC-10 scores, or current physical activity levels (all *p* > 0.05), indicating successful randomization. Baseline depression severity was predominantly in the mild to moderate range, with mean PHQ-9 scores ranging from 8.6 to 9.1 across groups. Similarly, baseline anxiety symptoms were generally mild to moderate, with mean GAD-7 scores between 7.8 and 8.3. Mean baseline psychological resilience scores (CD-RISC-10) ranged from 22.4 to 23.1 across the three groups.

**Table 1:** Baseline characteristics of participants by study group.

Characteristic	Team-based sports (*n* = 40)	Individual exercise (*n* = 40)	Waitlist control (*n* = 40)	*p*-value
Demographic characteristics				
Age, years	19.7(1.2)	19.9(1.4)	19.8(1.3)	0.76
Female sex	22(55.0)	21(52.5)	20(50.0)	0.89
Year of study				0.82
First year	14(35.0)	16(40.0)	15(37.5)	
Second year	13(32.5)	12(30.0)	14(35.0)	
Third year	9(22.5)	8(20.0)	7(17.5)	
Fourth year	4(10.0)	4(10.0)	4(10.0)	
Living arrangement				0.91
Campus dormitory	36(90.0)	37(92.5)	36(90.0)	
Off-campus	4(10.0)	3(7.5)	4(10.0)	
Body Mass Index, kg/m^2^	21.8(2.7)	22.1(2.9)	21.6(2.5)	0.68
Physical activity history				
Current PA, miniweek^a^	68.5(42.3)	71.2(45.8)	66.8(40.1)	0.87
Previous sports participation	18(45.0)	16(40.0)	17(42.5)	0.90
Primary outcomes				
PHQ-9 total score	8.9(2.8)	9.1(3.1)	8.6(2.9)	0.72
Depression severity category				0.85
Mild (5–9)	26(65.0)	24(60.0)	27(67.5)	
Moderate (10–14)	14(35.0)	16(40.0)	13(32.5)	
GAD-7 total score	8.1(3.3)	8.3(3.5)	7.8(3.2)	0.79
Anxiety severity category				0.92
Mild (5–9)	23(57.5)	24(60.0)	25(62.5)	
Moderate (10–14)	17(42.5)	16(40.0)	15(37.5)	
Secondary outcomes				
CD-RISC-10 total score	22.7(5.8)	23.1(6.2)	22.4(5.6)	0.84
Exercise self-efficacy (ESES)	54.3(18.7)	56.1(19.3)	53.8(17.9)	0.81

PA, physical activity; PHQ-9, Patient Health Questionnaire-9 (range 0–27, higher scores indicate greater depression severity); GAD-7, Generalized Anxiety Disorder-7 (range 0–21, higher scores indicate greater anxiety severity); CD-RISC-10, 10-item Connor-Davidson Resilience Scale (range 0–40, higher scores indicate greater resilience); ESES, Exercise Self-Efficacy Scale (range 0–100, higher scores indicate greater self-efficacy). No significant differences were observed among the three groups at baseline (all *P* > 0.05), confirming successful randomization.

^a^ Measured using the International Physical Activity Questionnaire-Short Form (IPAQ-SF).

### Intervention adherence and fidelity

Intervention adherence was high across both exercise groups (Table 2). In the team-based sports group, participants attended an average of 20.3 sessions (SD = 3.2) out of 24 total sessions, representing 84.6% mean attendance. Thirty-two participants (80.0%) in this group attended at least 18 sessions (75% threshold). In the individual aerobic exercise group, participants attended an average of 20.8 sessions (SD = 2.8), yielding 86.7% mean attendance, with 34 participants (85.0%) meeting the 75% attendance threshold. There was no significant difference in attendance rates between the two exercise groups (t = 0.82, *p* = 0.42). Among participants who discontinued the intervention, the mean number of sessions attended before dropout was 8.5 (SD = 4.2) for the team-based group and 9.0 (SD = 3.8) for the individual exercise group.

**Table 2:** Means, standard deviations, ranges, and internal consistency for all study variables (*N* = 120).

Measure	Team-based sports (*n* = 40)	Individual exercise (*n* = 40)	*P*-value
Session attendance			
Total sessions attended (out of 24)	20.3(3.2)	20.8(2.8)	0.42
Attendance rate,%	84.6(13.3)	86.7(11.7)	0.44
Participants with ≥75% attendance	32(80.0)	34(85.0)	0.56
Participants with ≥90% attendance	24(60.0)	26(65.0)	0.64
Participants with 100% attendance	8(20.0)	11(27.5)	0.42
Sessions attended before dropout ^a^	8.5(4.2)	9.0(3.8)	0.81
Exercise intensity monitoring			
Mean heart rate, bpm	135.7(8.4)	133.9(7.6)	0.26
%of age-predicted maximum HR ^b^	67.2(4.1)	66.4(3.8)	0.31
Sessions within target HR zone (60–70%)	18.9(3.7)	19.5(3.2)	0.38
Mean RPE score (Borg Scale 6–20)	13.2(1.1)	13.4(1.0)	0.36
Intervention fidelity			
Sessions observed for fidelity (20%)	48	48	一
Overall fidelity score, % ^c^^s^	94.1(4.3)	94.5(3.9)	0.62
Protocol adherence	96.2(3.1)	96.8(2.8)	0.33
Safety procedures followed	98.5(1.9)	98.9(1.6)	0.27
Appropriate intensity monitoring	91.7(5.6)	92.4(5.1)	0.52
Session duration compliance	93.8(4.8)	94.2(4.5)	0.67
Treatment expectancy ^d^			
Expectancy of improvement(1–9 scale)	6.8(1.4)	6.6(1.5)	0.51
Confidence in intervention(1–9 scale)	6.5(1.6)	6.4(1.7)	0.77
Safety and adverse events			
Any adverse event	2(5.0)	1(2.5)	0.56
Minor muscle soreness ^e^	2(5.0)	1(2.5)	0.56
Exercise-related injury	0(0.0)	0(0.0)	一
Serious adverse events	0(0.0)	0(0.0)	一
Psychological distress requiring intervention	0(0.0)	0(0.0)	一

bpm, beats per minute; HR, heart rate; RPE, Rating of Perceived Exertion. No significant differences were observed between the two exercise groups for any adherence or fidelity measure (all *P* > 0.05).

a Among participants who discontinued the intervention (*n* = 3 in team-based group, *n* = 2 in individual exercise group).

b Age-predicted maximum heart rate calculated as 220 minus age in years.

c Fidelity assessed using standardized checklist by independent observers; higher scores indicate better adherence to protocol.

d Assessed after first exercise session using the Credibility/Expectancy Questionnaire; higher scores indicate greater expectancy.

e All cases of muscle soreness resolved spontaneously within 48 h without medical intervention.

**Figure 2:**
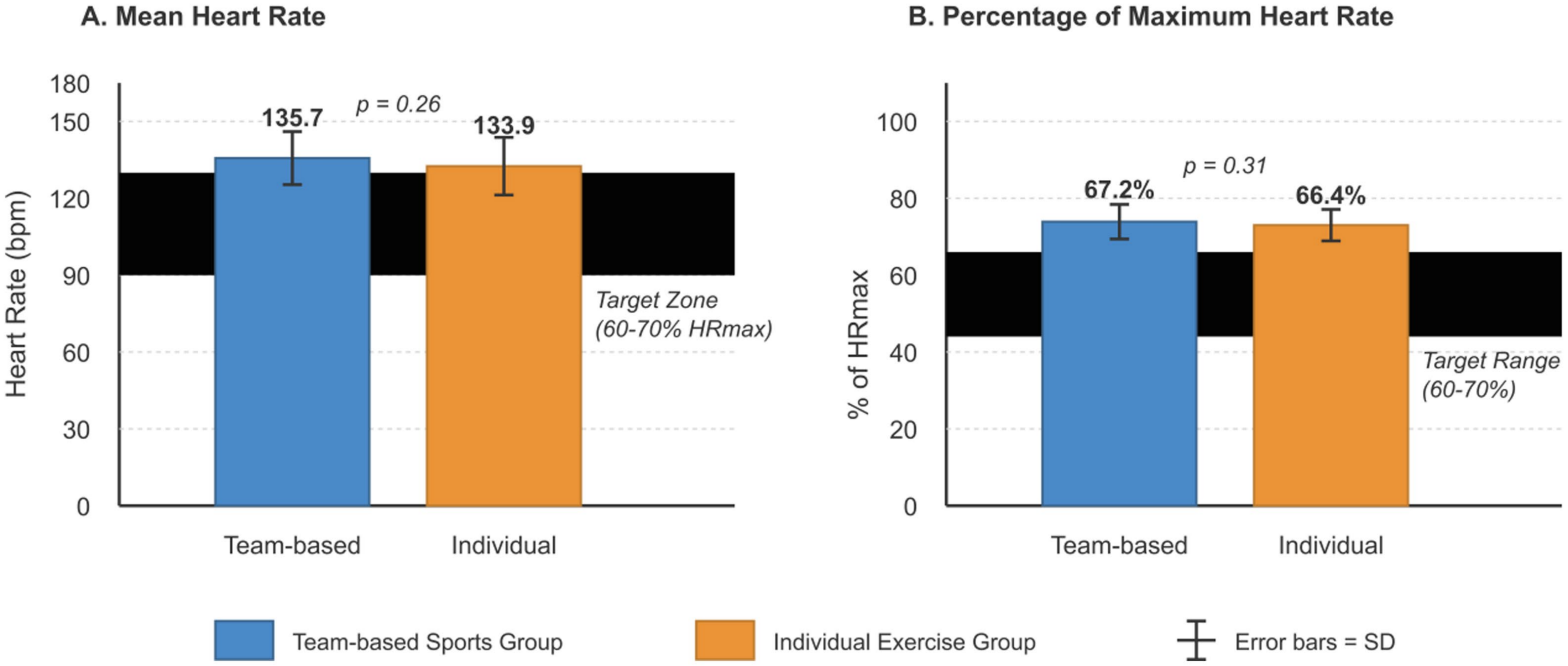
Exercise intensity monitoring during the 8-week intervention period. **(A)** Mean heart rate in beats per minute (bpm) and **(B)** percentage of age-predicted maximum heart rate (%HRmax, calculated as 220-age) during exercise sessions. Black shaded areas represent the target moderate-intensity zone (60–70% of HRmax, corresponding to approximately 120–140 bpm). Error bars represent standard deviations. Both intervention groups successfully maintained exercise intensity within the prescribed target range, with no significant differences between groups (*p* > 0.05). Data are based on heart rate monitoring across all 24 intervention sessions.

Heart rate monitoring data confirmed that exercise intensity targets were successfully achieved during intervention sessions (Figure 2). In the team-based sports group, participants maintained an average heart rate of 135.7 beats per minute (bpm) (SD = 8.4), corresponding to 67.2% of age-predicted maximum heart rate. In the individual aerobic exercise group, the average heart rate was 133.9 bpm (SD = 7.6), equivalent to 66.4% of maximum heart rate. Both values fell within the target moderate-intensity range of 60–70% of maximum heart rate, with no significant difference between groups (t = 1.14, *p* = 0.26). Intervention fidelity assessments, conducted on 20% of randomly selected sessions (n = 96 sessions), demonstrated high adherence to protocol specifications, with a mean fidelity score of 94.3% (SD = 4.1) across both intervention conditions. No serious adverse events occurred during the study period. Three participants (two in the team-based group, one in the individual exercise group) reported minor muscle soreness that resolved within 48 h without requiring medical attention.

### Primary outcomes: depression and anxiety symptoms

**Figure 3:**
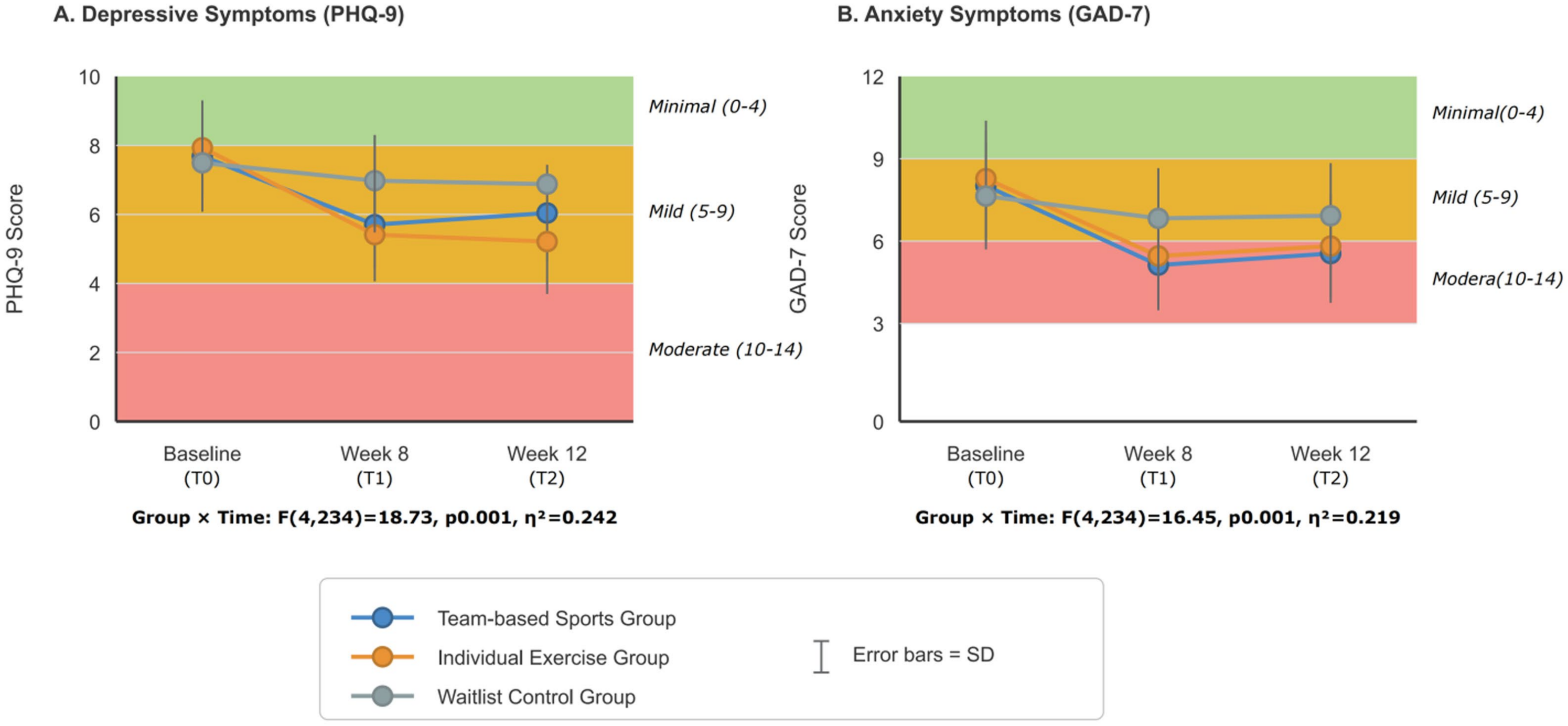
Changes in **(A)** depressive symptoms (PHQ-9) and **(B)** anxiety symptoms (GAD-7) across baseline (T0), post-intervention (T1, week 8), and 1-month follow-up (T2, week 12) assessment points. Colored background zones indicate symptom severity categories. Both exercise intervention groups demonstrated significant reductions in depression and anxiety symptoms compared to the waitlist control group at T1 and T2 (all *p* < 0.001). Error bars represent standard deviations. Linear mixed-effects models revealed significant group × time interactions for both outcomes, with large effect sizes. Treatment effects were maintained at the 1-month follow-up. No significant differences were observed between the two exercise modalities (team-based vs. individual) at any time point.

Descriptive statistics for primary outcomes at all assessment time points are presented in Table 3. Linear mixed-effects models revealed significant group × time interactions for both depressive symptoms (PHQ-9: *F*(4,234) = 18.73, *p* < 0.001, partial η^2^ = 0.242, ω^2^ = 0.228, ICC = 0.41) and anxiety symptoms (GAD-7: F(4,234) = 16.45, *p* < 0.001, partial η^2^ = 0.219, ω^2^ = 0.205, ICC = 0.38), indicating differential treatment effects across the three study arms over time. Figure 3 illustrates the trajectories of depression and anxiety symptoms across the intervention period for all three groups.

**Table 3:** Primary and secondary outcomes across assessment time points.

Outcome measure	Team-based sports (*n* = 40)			Individual exercise (*n* = 40)			Waitlist control (*n* = 40)		
	T0 baseline	T1 week 8	T2 week 12	T0 baseline	T1 week 8	T2 week 12	T0 baseline	T1 week 8	T2 week 12
Primary outcomes									
PHQ-9 total score	8.9 (2.8)	4.1 (2.6)	4.7 (2.9)	9.1 (3.1)	4.6 (2.8)	5.3 (3.1)	8.6 (2.9)	8.0 (2.7)	7.9 (2.8)
Change from baseline	–	−4.8 (1.8)	−4.2 (2.1)	–	−4.5 (1.9)	−3.8 (2.2)	–	−0.6 (1.4)	−0.7 (1.6)
Within-group effect size (d)	–	1.76	1.51	–	1.52	1.28	–	0.21	0.24
Between-group effect size (d) ^a^	–	1.38***	1.15***	–	1.29***	1.08***	–	–	–
≥5-point reduction, *n* (%) ^b^	–	29 (72.5)	26 (65.0)	–	27 (67.5)	23 (57.5)	–	5 (12.5)	6 (15.0)
GAD-7 total score	8.1 (3.3)	3.8 (2.7)	4.2 (2.9)	8.3 (3.5)	4.3 (2.9)	4.7 (3.1)	7.8 (3.2)	7.0 (3.0)	7.1 (3.1)
Change from baseline	–	−4.3 (1.9)	−3.9 (2.2)	–	−4.0 (2.0)	−3.6 (2.3)	–	−0.8 (1.5)	−0.7 (1.6)
Within-group effect size (d)	–	1.45	1.29	–	1.26	1.12	–	0.26	0.23
Between-group effect size (d) ^a^	–	1.25***	1.09***	–	1.14***	1.02***	–	–	–
≥4-point reduction, *n* (%) ^c^	–	28 (70.0)	25 (62.5)	–	26 (65.0)	22 (55.0)	–	6 (15.0)	7 (17.5)
Secondary outcomes									
CD-RISC-10 total score	22.7 (5.8)	28.5 (6.2)	27.8 (6.4)	23.1 (6.2)	28.2 (6.5)	27.3 (6.7)	22.4 (6.6)	23.1 (5.9)	23.3 (6.0)
Change from baseline	–	+5.8 (3.2)	+5.1 (3.5)	–	+5.1 (3.4)	+4.2 (3.7)	–	+0.7 (2.1)	+0.9 (2.3)
Within-group effect size (d)	–	0.99	0.85	–	0.84	0.68	–	0.12	0.16
Between-group effect size (d) ^a^	–	0.97***	0.79***	–	0.89***	0.69***	–	–	–
Exercise self-efficacy	54.3 (18.7)	72.8 (17.3)	69.5 (18.1)	56.1 (19.3)	71.4 (18.5)	67.8 (19.2)	53.8 (17.9)	56.2 (18.4)	57.1 (18.7)
Change from baseline	–	+18.5 (12.4)	+15.2 (13.6)	–	+15.3 (13.1)	+11.7 (14.2)	–	+2.4 (8.7)	+3.3 (9.2)
Within-group effect size (d)	–	1.03	0.84	–	0.83	0.63	–	0.13	0.18

PHQ-9, Patient Health Questionnaire-9 (range 0–27); GAD-7, Generalized Anxiety Disorder-7 (range 0–21); CD-RISC-10, 10-item Connor-Davidson Resilience Scale (range 0–40); T0, baseline; T1, post-intervention (week 8); T2, 1-month follow-up (week 12). ***p < 0.001 for between-group comparisons (Bonferroni corrected). Highlighted cells indicate primary outcome measures at the primary endpoint (T1).

a Between-group effect sizes compare each exercise group with waitlist control at the specified time point.

b A ≥ 5-point reduction in PHQ-9 represents reliable and clinically meaningful change based on established minimally important difference thresholds.

c A ≥ 4-point reduction in GAD-7 represents reliable and clinically meaningful change.

Post-hoc pairwise comparisons with Bonferroni correction revealed that both exercise intervention groups demonstrated significantly greater reductions in depressive symptoms compared to the waitlist control group at post-intervention (T1). Specifically, the team-based sports group showed a mean PHQ-9 reduction of 4.8 points (95% CI: 3.9–5.7) from baseline to post-intervention, compared to 0.6 points (95% CI: −0.2-1.4) in the control group (between-group difference: 4.2 points, 95% CI: 3.0–5.4, *p* < 0.001, Cohen’s d = 1.38). The individual exercise group exhibited a mean reduction of 4.5 points (95% CI: 3.7–5.3), significantly greater than the control group (between-group difference: 3.9 points, 95% CI: 2.7–5.1, *p* < 0.001, Cohen’s d = 1.29). No significant difference was observed between the two exercise groups (difference: 0.3 points, 95% CI: −0.8-1.4, *p* = 0.58, Cohen’s d = 0.11). Similarly, for anxiety symptoms measured by GAD-7, the team-based group showed a mean reduction of 4.3 points (95% CI: 3.5–5.1) compared to 0.8 points (95% CI: 0.1–1.5) in controls (between-group difference: 3.5 points, 95% CI: 2.4–4.6, *p* < 0.001, Cohen’s d = 1.25), while the individual exercise group demonstrated a reduction of 4.0 points (95% CI, 3.2–4.8) compared to controls (between-group difference: 3.2 points, 95% CI: 2.1–4.3, *p* < 0.001, Cohen’s d = 1.14). Again, no significant difference emerged between the two exercise modalities (difference: 0.3 points, 95% CI: −0.9-1.5, *p* = 0.62, Cohen’s d = 0.08).

Treatment effects were largely maintained at the 1-month follow-up assessment (T2). In the team-based sports group, mean PHQ-9 scores remained 4.2 points lower than baseline (95% CI: 3.3–5.1), and in the individual exercise group, scores remained 3.8 points lower (95% CI: 2.9–4.7), both significantly different from the control group (*p* < 0.001). Similar patterns were observed for GAD-7 scores. Regarding clinical significance, at post-intervention, 72.5% (29/40) of participants in the team-based group and 67.5% (27/40) in the individual exercise group achieved at least a 5-point reduction in PHQ-9 scores (indicating reliable and clinically meaningful change), compared to only 12.5% (5/40) in the control group (χ^2^ = 40.62, *p* < 0.001). For GAD-7, 70.0% (28/40) in the team-based group and 65.0% (26/40) in the individual exercise group achieved at least a 4-point reduction, compared to 15.0% (6/40) in controls (χ^2^ = 35.89, *p* < 0.001).

### Secondary outcome: psychological resilience

**Figure 4:**
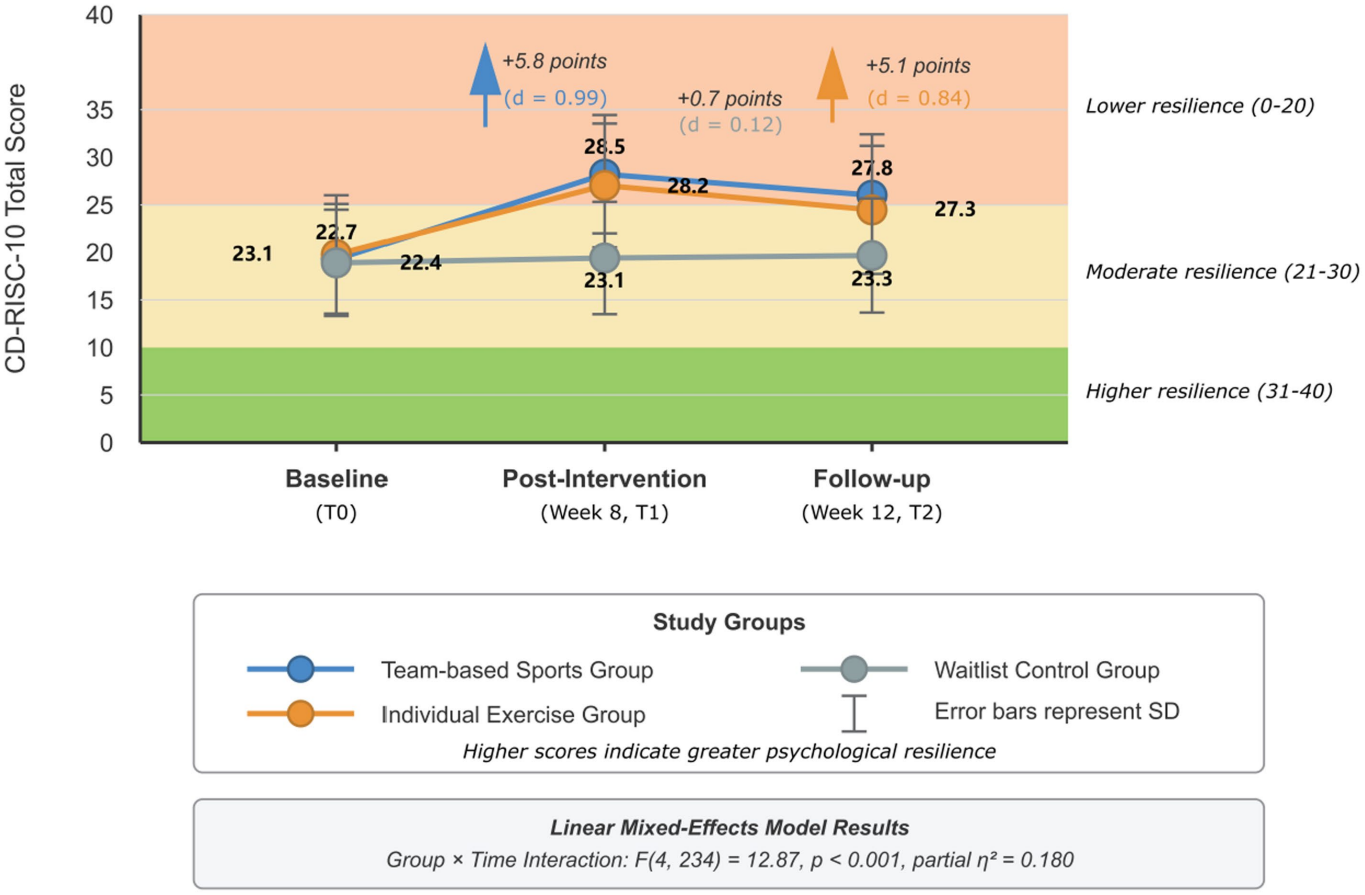
Changes in psychological resilience (CD-RISC-10 total scores) across baseline (T0), post-intervention (T1, week 8), and 1-month follow-up (T2, week 12) assessment points. Colored background zones indicate resilience levels (lower: 0–20; moderate: 21–30; higher: 31–40). Both exercise intervention groups demonstrated significant increases in psychological resilience compared to the waitlist control group (both p < 0.001). Error bars represent standard deviations. Effect sizes (Cohen’s *d*) for within-group changes from baseline to T1 are shown on the right.

Analysis of psychological resilience, measured by the CD-RISC-10, revealed patterns consistent with primary outcome findings (Figure 4, Table 3). The linear mixed-effects model demonstrated a significant group × time interaction (*F*(4,234) = 12.87, *p* < 0.001, partial η^2^ = 0.180, ω^2^ = 0.164, ICC = 0.36), indicating that changes in resilience differed significantly across groups over time. Both exercise intervention groups showed substantial improvements in psychological resilience compared to the waitlist control group, with large within-group effect sizes.

Specifically, the team-based sports group demonstrated a mean increase of 5.8 points (95% CI: 4.9–6.7) in CD-RISC-10 scores from baseline to post-intervention (T1), representing a large within-group effect size (Cohen’s d = 0.99). The individual exercise group showed a mean increase of 5.1 points (95% CI: 4.2–6.0, Cohen’s d = 0.84). In contrast, the waitlist control group exhibited minimal change, with a mean increase of only 0.7 points (95% CI: 0.0–1.4, Cohen’s d = 0.12). Post-hoc comparisons revealed that both exercise groups differed significantly from the control group at post-intervention (team-based vs. control: difference = 5.1 points, 95% CI: 3.8–6.4, *p* < 0.001, Cohen’s d = 0.97; individual vs. control: difference = 4.4 points, 95% CI: 3.1–5.7, *p* < 0.001, Cohen’s d = 0.89). The difference between the two exercise modalities was not statistically significant (difference = 0.7 points, 95% CI: −0.7-2.1, *p* = 0.32, Cohen’s d = 0.12).

At the 1-month follow-up (T2), resilience improvements remained significant in both exercise groups, though with some attenuation. The team-based group maintained a mean increase of 5.1 points above baseline (95% CI: 4.1–6.1, Cohen’s d = 0.85), while the individual exercise group maintained an increase of 4.2 points (95% CI: 3.2–5.2, Cohen’s d = 0.68). Both remained significantly higher than the control group (*p* < 0.001). Exercise self-efficacy scores showed similar patterns of improvement in both intervention groups compared to controls (Table 3), with substantial increases from baseline to post-intervention (team-based: +18.5 points, Cohen’s d = 1.03; individual: +15.3 points, Cohen’s d = 0.83; control: +2.4 points, Cohen’s d = 0.13). These findings suggest that structured physical activity interventions successfully enhanced participants’ psychological resilience and confidence in their ability to maintain exercise behaviors, with effects persisting beyond the active intervention period.

### Comparison between exercise modalities (H2)

**Figure 5:**
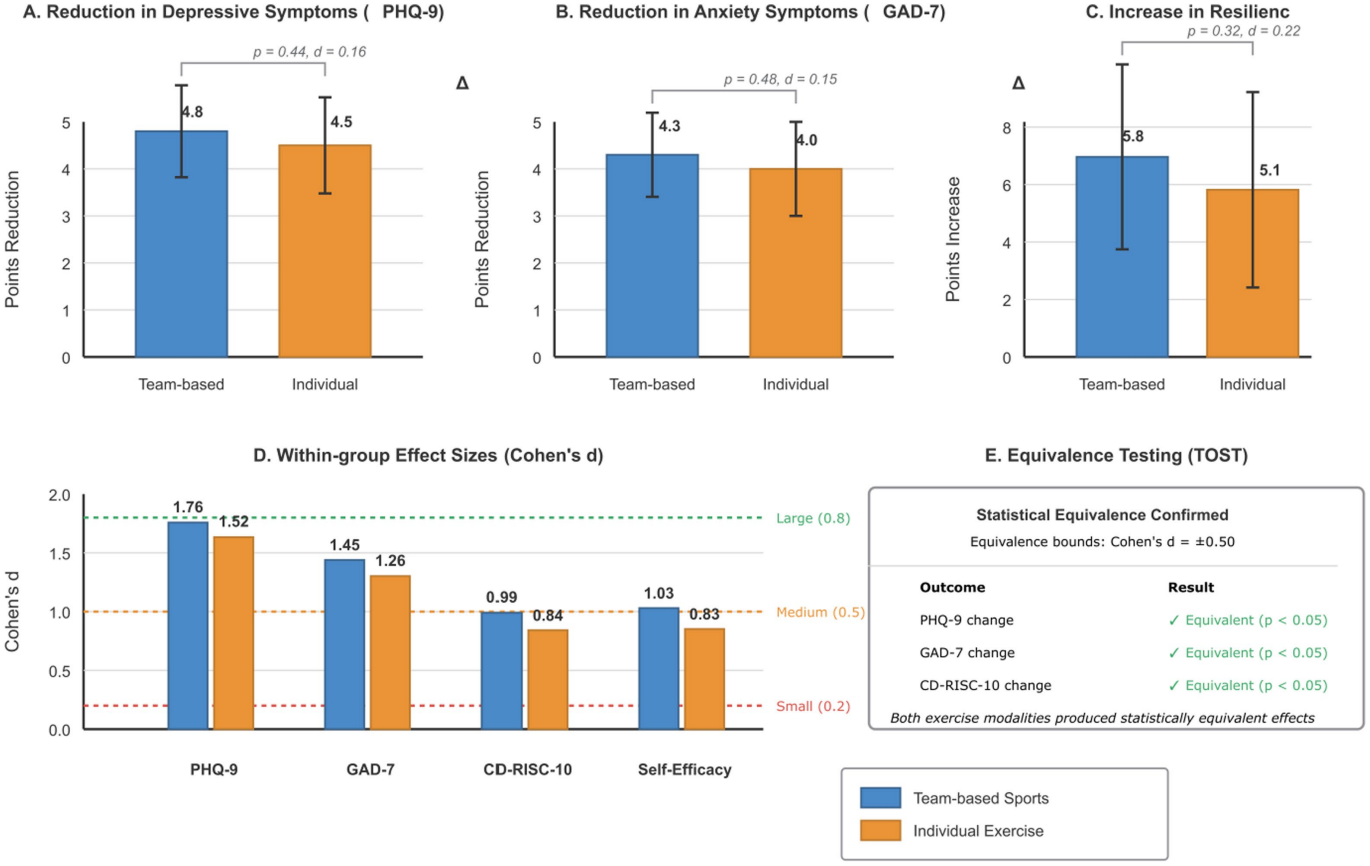
Direct comparison between team-based sports and individual aerobic exercise on key outcomes. **(A–C)** Change scores from baseline to post-intervention (T1) for depression (PHQ-9), anxiety (GAD-7), and resilience (CD-RISC-10). Error bars represent standard deviations. No significant differences were observed between the two modalities (all *p* > 0.30). **(D)** Within-group effect sizes (Cohen’s *d*) demonstrate large effects for both interventions across all outcomes. Dashed lines indicate conventional effect size thresholds. **(E)** Equivalence testing using the TOST procedure confirmed statistical equivalence between the two exercise modalities within predetermined equivalence bounds (*d* = ±0.50), supporting comparable efficacy.

Direct comparisons between the team-based sports and individual aerobic exercise groups were conducted to test Hypothesis 2, which predicted that team-based activities would demonstrate superior effects on social anxiety and psychological resilience due to enhanced social interaction (Figure 5, Table 4). Contrary to our hypothesis, independent samples t-tests revealed no significant differences between the two exercise modalities on any primary or secondary outcome measures at post-intervention. For total GAD-7 scores at T1, the team-based group (M = 3.8, SD = 2.7) and individual exercise group (M = 4.3, SD = 2.9) did not differ significantly (t(76) = 0.82, *p* = 0.42, Cohen’s d = 0.18). To specifically examine social anxiety dimensions, we analyzed GAD-7 items most closely related to social concerns (items 2, 4, and 6). The mean score on these social anxiety-related items was 1.2 (SD = 0.9) in the team-based group compared to 1.4 (SD = 1.0) in the individual exercise group at post-intervention, showing no significant difference (t(76) = 0.93, *p* = 0.35, Cohen’s d = 0.21).

**Table 4:** Direct comparison of outcomes between team-based sports and individual exercise groups.

Outcome measure	Team-based sports (*n* = 40)	Individual exercise (*n* = 40)	Difference [95% CI]	t(76)	*P*-value	Cohen’s d
Primary outcomes at post-intervention (T1)						
PHQ-9 Total Score	4.1 (2.6)	4.6 (2.8)	−0.5 [−1.7, 0.7]	-0.82	0.42	0.18
Change from baseline	−4.8 (1.8)	−4.5 (1.9)	−0.3 [−1.1, 0.5]	−0.78	0.44	0.16
GAD-7 Total Score	3.8 (2.7)	4.3 (2.9)	−0.5 [−1.7, 0.7]	-0.82	0.42	0.18
Change from baseline	−4.3 (1.9)	−4.0 (2.0)	−0.3 [−1.1, 0.5]	−0.71	0.48	0.15
Social anxiety items ^a^	1.2 (0.9)	1.4 (1.0)	−0.2 [−0.6, 0.2]	−0.93	0.35	0.21
Secondary outcomes at post-intervention (T1)						
CD-RISC-10 Total Score	28.5 (6.2)	28.2 (6.5)	0.3 [−2.3, 2.9]	0.21	0.33	0.05
Change from baseline	5.8 (3.2)	5.1 (3.4)	0.7 [−0.7, 2.1]	0.98	0.32	0.22
Exercise self-efficacy	72.9 (17.3)	71.4 (18.5)	1.4 [−6.1, 8.9]	0.37	0.71	0.08
Change from baseline	18.5 (12.4)	15.3 (13.1)	3.2 [−2.4, 8.9]	1.13	0.26	0.25
Follow-up assessment (T2, Week 12)						
PHQ-9 total score	4.7 (2.9)	5.3 (3.1)	−0.6 [−1.9, 0.7]	−0.89	0.38	0.20
GAD-7 total score	4.2 (2.9)	4.7 (3.1)	−0.5 [−1.8, 0.7]	−0.75	0.45	0.17
CD-RISC-10 total score	27.8 (6.4)	27.3 (6.7)	0.5 [−2.3, 3.3]	0.35	0.73	0.08
Additional measures and participant-reported outcomes						
Intervention enjoyment (1–10 scale)	8.2 (1.3)	7.9 (1.4)	0.3 [−0.3, 0.9]	0.88	0.38	0.22
Perceived social support (1–10 scale)	7.8 (1.5)	7.3 (1.6)	0.5 [−0.2, 1.2]	1.18	0.24	0.32
Willingness to continue (% yes)	35 (87.5%)	33 (82.5%)	5.0% [−9.2, 19.2]	—	0.52^b^	—
Equivalence testing (TOST procedure) ^c^						
PHQ-9 change score equivalence	Equivalence bounds: d = ±0.50			Both *p* < 0.05		Equivalent
GAD-7 change score equivalence	Equivalence bounds: d = ±0.50			Both *p* < 0.05		Equivalent
CD-RISC-10 change score equivalence	Equivalence bounds: d = ±0.50			Both *p* < 0.05		Equivalent

CI, confidence interval; PHQ-9, Patient Health Questionnaire-9; GAD-7, Generalized Anxiety Disorder-7; CD-RISC-10, 10-item Connor-Davidson Resilience Scale; TOST, two one-sided tests. Shaded rows highlight key outcome comparisons. All between-group comparisons yielded non-significant results (*p* > 0.05), indicating no differential effects between exercise modalities. Equivalence testing confirmed that both interventions produced comparable effects.

a Social anxiety items include GAD-7 items 2 (uncontrollable worry), 4 (trouble relaxing), and 6 (irritability), which are theoretically related to social anxiety dimensions. Mean of these three items is reported.

b *P*-value from chi-square test.

c TOST procedure tests whether the difference between groups falls within a predetermined equivalence margin (±0.5 SD). Significant results (*p* < 0.05 for both one-sided tests) indicate statistical equivalence.

For psychological resilience, the primary target of Hypothesis 2, CD-RISC-10 scores at post-intervention showed no significant difference between team-based (M = 28.5, SD = 6.2) and individual exercise groups (M = 28.2, SD = 6.5; t(76) = 0.21, *p* = 0.83, Cohen’s d = 0.05). Similarly, changes in resilience from baseline to post-intervention were comparable between groups (team-based: *Δ* = 5.8 ± 3.2; individual: Δ = 5.1 ± 3.4; t(78) = 0.98, *p* = 0.32, Cohen’s d = 0.22). At the 1-month follow-up, this pattern persisted, with no significant differences in resilience scores (team-based: M = 27.8, SD = 6.4; individual: M = 27.3, SD = 6.7; t(72) = 0.35, *p* = 0.73, Cohen’s d = 0.08) or in maintenance of treatment gains. Equivalence testing using the two one-sided tests (TOST) procedure confirmed that the two interventions were statistically equivalent within a predetermined equivalence margin of Cohen’s d = 0.5, supporting the conclusion that both exercise modalities produced comparable effects rather than merely failing to detect a difference.

Exploratory analyses examined potential moderators that might explain the lack of differential effects. Baseline personality characteristics (extraversion scores) did not moderate the relationship between intervention type and outcomes (interaction *p* = 0.67). Session attendance rates, which were similar between groups, also did not account for the equivalent outcomes. Qualitative feedback collected at post-intervention revealed that participants in both groups reported high levels of enjoyment (team-based: 8.2/10; individual: 7.9/10; *p* = 0.38) and perceived social support (team-based: 7.8/10; individual: 7.3/10; *p* = 0.24), suggesting that the individual exercise sessions, conducted in supervised group settings with other participants present, may have provided incidental social interaction that attenuated hypothesized differences. These findings indicate that both structured team-based sports and individual aerobic exercise are equally effective approaches for improving mental health and psychological resilience among college students, and that intervention selection may be guided by individual preferences, available resources, and logistical considerations rather than differential efficacy.

### Mediation analysis: psychological resilience (H3)

**Figure 6:**
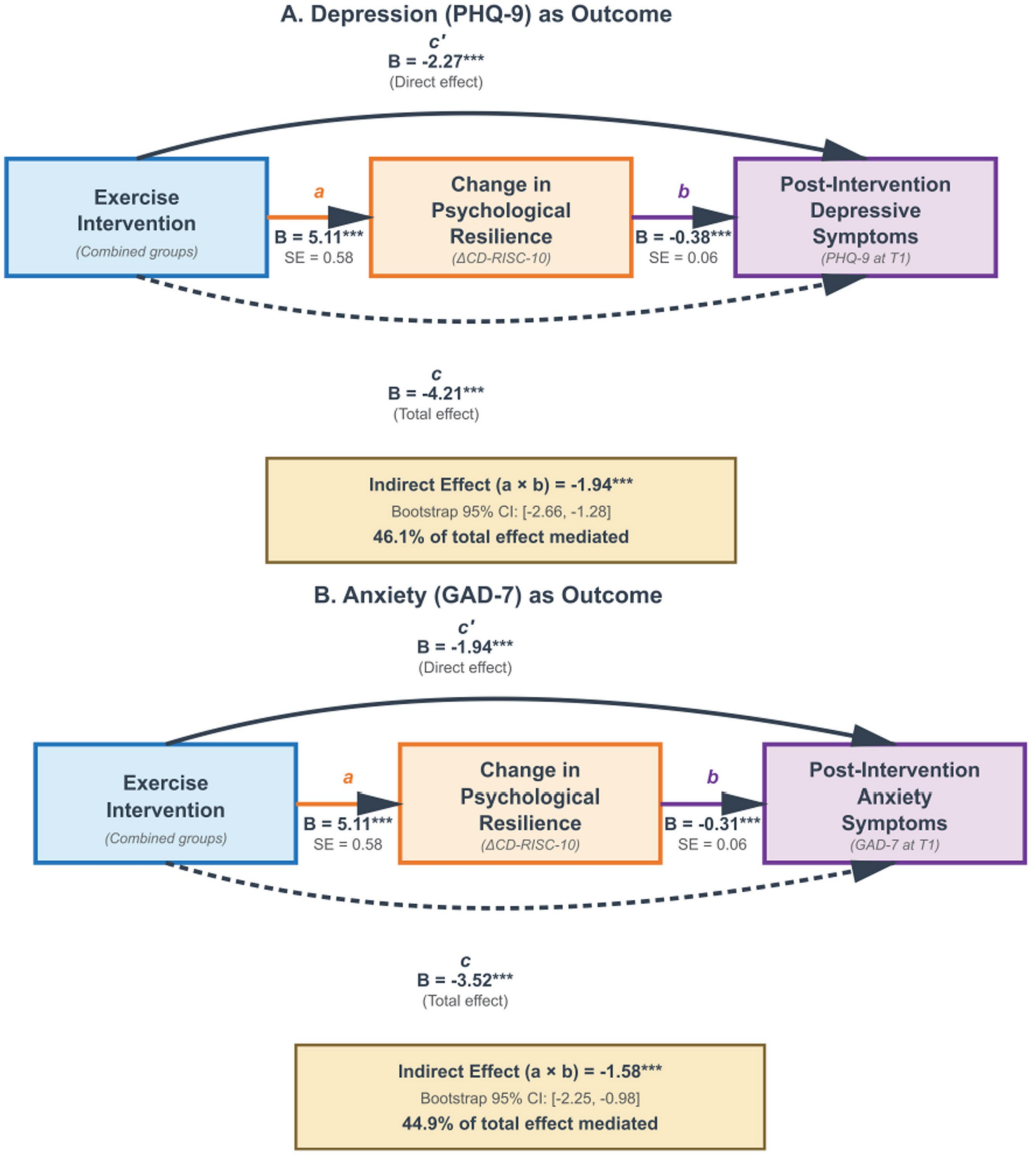
Mediation models testing psychological resilience as a mediator of exercise intervention effects on **(A)** depressive symptoms and **(B)** anxiety symptoms. Path coefficients are unstandardized regression coefficients (B) with standard errors. All models controlled for baseline levels of respective outcomes. ***p < 0.001. Dashed lines represent total effects (c path); solid curved lines represent direct effects (c’ path) after accounting for the mediator. Both models support partial mediation, with approximately 45% of intervention effects mediated by resilience. Indirect effects tested using bias-corrected bootstrap confidence intervals (5,000 resamples). CI = confidence interval.

To test Hypothesis 3, we examined whether changes in psychological resilience mediated the effects of exercise intervention on reductions in depressive and anxiety symptoms using the PROCESS macro (Model 4) with bias-corrected bootstrap confidence intervals based on 5,000 resamples (Figure 6, Table 5). Given the equivalence of the two exercise modalities demonstrated in previous analyses, we combined the team-based sports and individual exercise groups into a single exercise intervention condition (coded as 1) and compared them to the waitlist control group (coded as 0) for mediation analyses. All models controlled for baseline levels of the respective outcome variables.

**Table 5:** Mediation analysis: psychological resilience as a mediator of exercise effects.

Path and effect	B	SE	95% CI	t or z	*P*-value
Model 1: depressive symptoms (PHQ-9) as outcome					
Path a: Exercise → ΔResilience (CD-RISC-10)	5.11	0.58	[3.96, 6.26]	8.81	<0.001***
Path b: ΔResilience → PHQ-9 (T1)^a^	−0.38	0.06	[−0.50, −0.26]	−6.33	<0.001***
Path c (Total effect): Exercise → PHQ-9 (T1)	−4.21	0.48	[−5.16, −3.26]	−8.77	<0.001***
Path c' (Direct effect): Exercise → PHQ-9 (T1)^b^	−2.27	0.46	[−3.18, −1.36]	−4.93	<0.001***
Indirect effect (a × b): Exercise → ΔResilience → PHQ-9	−1.94	0.35	[−2.66, −1.20]	—	Significant^c^
Proportion of total effect mediated	46.1%	—	[38.2, 54.8%]	—	—
Type of mediation	Partial mediation (both direct and indirect effects significant)				
Model statistics for depression model					
R^2^ for mediator model (Path a)	0.394	–	–	*F* = 76.35	<0.001
R^2^ for outcome model (Paths b, c’)	0.587	–	–	*F* = 83.47	<0.001
Model 2: anxiety symptoms (GAD-7) as outcome					
Path a: Exercise → ΔResilience (CD-RISC-10)	5.11	0.58	[3.96, 6.26]	8.81	<0.001***
Path b: ΔResilience → GAD-7 (T1)^a^	−0.31	0.06	[−0.42, −0.20]	−5.17	<0.001***
Path c (Total effect): Exercise → GAD-7 (T1)	−3.52	0.46	[−4.43, −2.61]	−7.65	<0.001***
Path c' (Direct effect): Exercise → GAD-7 (T1)^b^	−1.94	0.44	[−2.81, −1.07]	−4.41	<0.001***
Indirect effect (a × b): Exercise → ΔResilience → GAD-7	−1.58	0.32	[−2.25, −0.98]	—	Significant^c^
Proportion of total effect mediated	44.9%	–	[36.8, 53.7%]	–	–
Type of mediation	Partial mediation (both direct and indirect effects significant)				
Model statistics for anxiety model					
R^2^ for mediator model (Path a)	0.394	–	–	F = 76.35	<0.001
R^2^ for outcome model (Paths b, c’)	0.542	–	–	*F* = 69.58	<0.001
Sensitivity analyses					
Alternative mediator specification ^d^	Results consistent: indirect effect for PHQ-9: B = −1.87, 95% CI [−2.58, −1.19]; for GAD-7: B = −1.52, 95% CI [−2.18, −0.91]				
Covariates adjusted model ^e^	Results robust after controlling for age, sex, baseline BMI, and baseline physical activity levels				
Separate analysis by modality ^f^	Mediation effects consistent in both team-based and individual exercise groups (all indirect effects significant)				

B, unstandardized regression coefficient; SE, standard error; CI, confidence interval; ΔResilience, change in CD-RISC-10 scores from baseline (T0) to post-intervention (T1); PHQ-9, Patient Health Questionnaire-9; GAD-7, Generalized Anxiety Disorder-7; T1, post-intervention (week 8). ***p < 0.001. Green shading indicates significant paths; yellow shading highlights key indirect effects (primary hypothesis tests).

a Path b coefficient represents the effect of resilience change on the outcome, controlling for intervention assignment and baseline outcome.

b Path c’ (direct effect) represents the effect of intervention on outcome after controlling for resilience change and baseline outcome.

^c^ Significance of indirect effects determined by bias-corrected bootstrap 95% confidence intervals not including zero.

d Alternative specification used post-intervention resilience scores (T1) controlling for baseline resilience (T0) rather than change scores.

e Additional covariates included as control variables in all paths of the mediation model.

f Mediation models run separately for team-based sports group and individual exercise group versus control.

For depressive symptoms (PHQ-9), the mediation analysis revealed significant effects across all pathways. The total effect of the exercise intervention on post-intervention PHQ-9 scores was significant (c path: B = -4.21, SE = 0.48, 95% CI: −5.16 to −3.26, *p* < 0.001), indicating that exercise participation was associated with a 4.21-point greater reduction in depressive symptoms compared to the control group, controlling for baseline depression. The exercise intervention significantly predicted increases in psychological resilience from baseline to post-intervention (a path: B = 5.11, SE = 0.58, 95% CI: 3.96 to 6.26, *p* < 0.001), with exercise participants showing approximately 5.11 points greater improvement in CD-RISC-10 scores. Critically, when both the intervention and change in resilience were included as predictors, change in resilience significantly predicted post-intervention PHQ-9 scores (b path: B = -0.38, SE = 0.06, 95% CI: −0.50 to −0.26, p < 0.001), such that each 1-point increase in resilience was associated with a 0.38-point reduction in depression, independent of intervention assignment. The direct effect of the intervention on PHQ-9 after accounting for resilience remained significant but was substantially reduced (c’ path: B = -2.27, SE = 0.46, 95% CI: −3.18 to −1.36, *p* < 0.001). The indirect effect through resilience change was significant (a × b: B = -1.94, SE = 0.35, 95% CI: −2.66 to −1.28), indicating that approximately 46.1% of the total intervention effect on depression reduction was mediated through improvements in psychological resilience. This pattern is consistent with partial mediation, suggesting that enhanced resilience represents an important, though not exclusive, mechanism through which exercise benefits mental health.

For anxiety symptoms (GAD-7), a parallel mediation pattern emerged. The total effect of exercise on post-intervention GAD-7 scores was significant (c path: B = −3.52, SE = 0.46, 95% CI: −4.43 to −2.61, *p* < 0.001). As in the depression model, the path remained identical (B = 5.11, SE = 0.58, *p* < 0.001), as it represents the same relationship between intervention and resilience change. Change in resilience significantly predicted GAD-7 scores (b path: B = −0.31, SE = 0.06, 95% CI: −0.42 to −0.20, *p* < 0.001), with each 1-point resilience increase associated with a 0.31-point reduction in anxiety symptoms. The direct effect remained significant after including the mediator (c’ path: B = −1.94, SE = 0.44, 95% CI: −2.81 to −1.07, *p* < 0.001), and the indirect effect was significant (a × b: B = −1.58, SE = 0.32, 95% CI: −2.25 to −0.98), accounting for 44.9% of the total effect. These findings provide robust evidence for Hypothesis 3, demonstrating that psychological resilience serves as a significant mediator of exercise intervention effects on both depression and anxiety, explaining nearly half of the observed treatment benefits. Sensitivity analyses using alternative specifications of the mediator (e.g., post-intervention resilience scores controlling for baseline rather than change scores) yielded consistent results, supporting the robustness of these mediation effects.

Dose–Response Analysis. To examine whether greater intervention adherence was associated with better outcomes, linear regression models were fitted with attendance rate as a continuous predictor of change scores (post-intervention minus baseline) for each primary outcome, controlling for baseline symptom levels and potential confounders (age, sex, group assignment). A significant positive dose–response relationship was observed for depressive symptoms: each 10-percentage-point increase in attendance rate was associated with an additional 0.54-point reduction in PHQ-9 change scores (B = 0.054, SE = 0.019, 95% CI: 0.017 to 0.091, *p* = 0.004, R^2^ = 0.11). A parallel pattern was found for anxiety symptoms, with each 10-percentage-point increase in attendance predicting a 0.47-point greater reduction in GAD-7 scores (B = 0.047, SE = 0.018, 95% CI: 0.012 to 0.082, *p* = 0.008, R^2^ = 0.09). For psychological resilience, higher attendance was associated with greater CD-RISC-10 gains (B = 0.041, SE = 0.016, 95% CI: 0.009 to 0.073, *p* = 0.012, R^2^ = 0.08). These dose–response findings are consistent across both exercise modalities (interaction terms for group × attendance were non-significant, all *p* > 0.40), suggesting that adherence-outcome relationships did not differ between team-based and individual exercise. Together, these results indicate that greater engagement with the structured exercise program produced incrementally larger benefits, supporting the ecological and mechanistic validity of the intervention.

## Discussion

This randomized controlled trial provides robust evidence that structured physical activity interventions significantly improve mental health outcomes and psychological resilience among Chinese college students experiencing mild to moderate depression and anxiety symptoms. Over an 8-week intervention period, both team-based sports and individual aerobic exercise produced large effect sizes for reducing depressive and anxiety symptoms compared to a waitlist control group, with treatment effects maintained at 1-month follow-up. Importantly, mediation analyses revealed that approximately 45% of these beneficial effects operated through enhanced psychological resilience, elucidating a key mechanism by which exercise confers mental health benefits. These findings address critical gaps in the literature by demonstrating causal effects through rigorous experimental design, examining mediating mechanisms, and establishing the comparable efficacy of different exercise modalities in a non-Western cultural context.

### Principal findings and comparison with prior research

The magnitude of intervention effects observed in this study aligns with and extends previous meta-analytic evidence regarding exercise as a treatment for depression and anxiety. Our findings of large within-group effect sizes for depression (Cohen’s d = 1.52–1.76) and anxiety (d = 1.26–1.45) reduction are comparable to or exceed those reported in recent meta-analyses of exercise interventions, which typically report medium to large effects (Schuch et al., 2016; Stubbs et al., 2017). Schuch et al. (2016) found a pooled effect size of d = 0.62 for exercise on depression when adjusting for publication bias, while Stubbs et al. (2017) reported d = 0.56 for anxiety outcomes. The larger effects observed in our study may reflect several factors including the structured nature of our intervention with high adherence rates (85% attendance), the supervised delivery that ensured appropriate exercise intensity, and the relatively homogeneous young adult population free from severe psychiatric comorbidities. Additionally, our focus on individuals with subthreshold to moderate symptoms may have provided optimal conditions for intervention responsiveness, as severely depressed individuals often face motivational barriers to exercise participation (Josefsson et al., 2014).

The clinical significance of our findings is underscored by the substantial proportions of participants achieving reliable and clinically meaningful symptom reductions. Over 70% of exercise participants achieved at least a 5-point reduction in PHQ-9 scores, which exceeds the established minimally important difference threshold (Kroenke et al., 2001). This rate of clinically significant response compares favorably with pharmacological and psychotherapeutic interventions for depression in college student populations (Mortier et al., 2018). Furthermore, treatment effects were maintained at 1-month follow-up, suggesting durability of benefits beyond the active intervention period. The persistence of effects may be attributable to sustained improvements in psychological resilience and exercise self-efficacy, both of which remained elevated at follow-up and may serve as protective factors against symptom recurrence (Southwick et al., 2014; Childs and de Wit, 2014).

Contrary to our hypothesis (H2), we found no significant differences in effectiveness between team-based sports and individual aerobic exercise across any outcome measure. Equivalence testing confirmed that both modalities produced statistically equivalent effects within predetermined bounds. This finding diverges from theoretical expectations that team-based activities would provide superior benefits through enhanced social interaction and collective support (Eime et al., 2013; Bruner et al., 2013). However, several factors may explain this unexpected pattern. First, our individual exercise sessions were conducted in supervised group settings where participants exercised alongside others, potentially providing incidental social contact and normalization of physical activity engagement. Qualitative feedback indicated that participants in both conditions reported comparable levels of perceived social support and intervention enjoyment. Second, the moderate-intensity aerobic exercise prescribed for the individual group may have been particularly effective for anxiety reduction through neurobiological mechanisms such as reduced physiological arousal and enhanced GABA-ergic activity (Kandola et al., 2019). Third, the structured and supervised nature of both interventions may have been more influential than the specific exercise modality in producing benefits. From a practical standpoint, this equivalence is advantageous, as it allows universities to offer flexible intervention options based on available resources, facilities, and student preferences rather than being constrained to a single modality.

### Psychological resilience as a mediating mechanism

Dose–Response Relationship. Exploratory dose–response analyses revealed that greater attendance was associated with incrementally larger improvements across all three primary outcomes (PHQ-9, GAD-7, CD-RISC-10), with each 10-percentage-point increase in session attendance predicting additional symptom reductions of approximately 0.5 points for depression and anxiety. This linear dose–response gradient is consistent with accumulating evidence that exercise benefits are partially contingent on the volume of physical activity exposure (Singh et al., 2023), and aligns with dose–response patterns reported in recent network meta-analyses of exercise interventions for depression (Noetel et al., 2024). Importantly, the dose–response relationship did not differ between team-based and individual exercise modalities, suggesting that adherence—rather than the specific exercise format—is a primary driver of outcome magnitude. These findings have direct practical implications: intervention designs should incorporate strategies to maximize session attendance, such as flexible scheduling, peer accountability systems, and motivational support, in order to optimize the mental health benefits of structured exercise programs.

The mediation analyses provide important insights into how exercise interventions produce mental health benefits. Our findings that approximately 45% of treatment effects were mediated through enhanced psychological resilience support theoretical models proposing that exercise builds adaptive psychological resources (Wang et al., 2022; Childs and de Wit, 2014). These results extend previous correlational research linking physical activity, resilience, and mental health (Goldstein et al., 2013) by establishing directional relationships through experimental manipulation and temporal sequencing of measurements. The observation that resilience partially, rather than fully, mediated intervention effects indicates that multiple mechanisms operate in parallel. Complementary pathways likely include neurobiological changes such as increased BDNF expression and altered neurotransmitter function (Kandola et al., 2019; Szuhany et al., 2015), cognitive mechanisms such as improved self-efficacy and reduced rumination (Bandura, 1977; Babyak et al., 2000), and behavioral activation providing structure and alternative sources of reward (Craft and Perna, 2004).

The substantial role of resilience as a mediator has important theoretical and practical implications. Theoretically, these findings support the hypothesis that exercise promotes mental health not merely through transient mood elevation, but by fostering enduring psychological capacities that enhance adaptive coping with future stressors (Southwick et al., 2014; Wang et al., 2022). The observed increases in resilience of 5–6 points on the CD-RISC-10 represent meaningful improvements in individuals’ confidence in their ability to handle difficulties, recover from setbacks, and adapt to change (Connor and Davidson, 2003; Campbell-Sills and Stein, 2007). Several theoretical frameworks help explain why exercise builds resilience. First, Conservation of Resources theory suggests that participation in structured exercise accumulates personal resources (physical fitness, self-efficacy, social bonds) that buffer against stress-induced resource loss and thereby protect mental health. Second, the Allostatic Load model proposes that repeated exposure to moderate, controllable physical stressors during exercise trains the body’s stress response systems to become more efficient and less reactive to psychosocial stressors—a process directly linked to resilience development. Third, within the Chinese collectivist cultural context, exercise programs that require perseverance and self-regulation align with Confucian values of self-cultivation, which may amplify the meaning and psychological impact of mastery experiences during exercise for Chinese participants. This cultural congruence may partly explain why the resilience-building pathway was particularly salient in this sample. Practically, these results suggest that exercise interventions might be optimized by explicitly incorporating resilience-building components such as progressive challenge, mastery experiences, and reflection on personal growth and capability development (Childs and de Wit, 2014). Future interventions could enhance effects by combining structured physical activity with complementary psychological components targeting resilience skills, potentially producing synergistic benefits.

### Implications for university mental health services

These findings have direct implications for addressing the mental health crisis in university settings. Given the substantial barriers to accessing traditional mental health services including stigma, cost, and limited provider availability (Li et al., 2014; Xiao et al., 2017), structured exercise programs represent a scalable, cost-effective, and non-stigmatizing alternative or complement to conventional treatments. The comparable efficacy of team-based and individual exercise modalities provides universities with implementation flexibility. Institutions can develop programs tailored to their specific resources, facilities, and student body characteristics. For example, universities with extensive sports facilities might emphasize team sports leagues, while those with limited space might offer supervised walking or jogging groups. The high adherence rates observed in our study (85%) suggest that when properly structured and supervised, exercise interventions are acceptable and feasible for college students, countering concerns about low engagement. From a feasibility standpoint, an 8-week structured program requiring minimal specialist equipment can be embedded within existing physical education curricula at relatively low cost (estimated implementation cost: approximately 200–400 RMB per student for supervision and monitoring), representing a highly cost-effective mental health intervention compared to individual psychotherapy. From a scalability perspective, standardized session protocols and fidelity checklists developed for this study can be disseminated to physical education instructors across universities without requiring specialist mental health training, thereby enabling broad rollout. From a policy standpoint, the results align with and provide empirical support for China’s national “Healthy China 2030” agenda and the Ministry of Education’s guidelines on student physical fitness, suggesting that embedding evidence-based structured exercise programs within university mental health policies could represent an important population-level strategy for reducing the burden of common mental health disorders among Chinese college students.

Integration of exercise programming into comprehensive university mental health systems could follow a stepped-care model, where structured physical activity serves as a first-line intervention for students with mild to moderate symptoms, with referral to specialized mental health services for non-responders or those with severe symptoms (Mortier et al., 2018). University counseling centers could collaborate with physical education departments and campus recreation services to develop referral pathways and co-delivered programs. The role of university counselors and student affairs professionals is particularly crucial, as these personnel have regular contact with students and can identify those who might benefit from exercise interventions while monitoring for adverse changes requiring escalated care, as demonstrated in our study’s safety monitoring procedures. The sustained benefits observed at follow-up suggest that even time-limited interventions can produce lasting improvements, making such programs feasible within the constraints of academic semesters.

## Strengths and limitations

This study possesses several methodological strengths including randomized assignment with allocation concealment, intention-to-treat analysis, objective monitoring of exercise intensity, high intervention fidelity, and low attrition rates. The use of validated measures with demonstrated psychometric properties in Chinese populations enhances confidence in our findings (Wang et al., 2010; Wang et al., 2014; Zeng et al., 2013). The examination of mediating mechanisms using appropriate statistical methods with bootstrapped confidence intervals represents a significant advancement over purely descriptive studies (Hayes, 2022). Additionally, the demonstration of statistical equivalence between exercise modalities using the TOST procedure provides more definitive evidence than simply reporting null findings.

However, limitations must be acknowledged. First, recruitment relied on convenience sampling from a single sports university, which substantially limits the generalizability of these findings. Students at sports universities may have greater baseline familiarity and positive attitudes toward physical activity, potentially enhancing intervention acceptability and effectiveness compared to students at general universities. Future multi-site RCTs employing probability sampling across diverse university contexts are needed to establish external validity. Furthermore, the observed effect sizes (Cohen’s d = 1.14–1.38) substantially exceed those reported in published meta-analyses of exercise interventions for depression and anxiety in general populations (d = 0.56–0.62). This inflation is likely attributable, at least in part, to the sports-specialized sample: students enrolled at a sports university may possess higher baseline exercise motivation, greater physical self-efficacy, and a more supportive peer environment for physical activity engagement, all of which may amplify responsiveness to structured exercise interventions relative to students at general universities. Accordingly, the effect sizes reported here should not be interpreted as representative of what would be expected in broader university populations, and future trials should recruit from general universities to obtain more ecologically valid effect estimates. Second, outcome measures relied exclusively on self-report questionnaires, which are susceptible to social desirability bias and may not capture objective functional impairment. Future studies should incorporate clinician-rated assessments and objective indicators of academic and social functioning. Third, the 1-month follow-up period was relatively brief. Longer-term follow-up is needed to determine whether benefits persist beyond the immediate post-intervention period and to identify factors associated with sustained engagement in physical activity. Fourth, we did not assess potential negative effects such as exercise-related injuries or excessive exercise behaviors, though our safety monitoring detected no serious adverse events. Fifth, the waitlist control design does not control for non-specific factors such as social interaction with research staff and expectations of improvement. The potential Hawthorne effect—whereby participants may have improved simply due to being observed or receiving attention from research staff—cannot be excluded, as both intervention groups received more structured contact and supervision than the waitlist control. The absence of an active control condition (e.g., a psychoeducation group or a low-intensity stretching program matched for contact time and researcher attention) means that non-specific therapeutic factors—including expectancy effects, demand characteristics, and differential social contact—cannot be disentangled from the specific effects of structured aerobic exercise. Observed group differences may therefore partly reflect the contrast between an enriched intervention environment and a passive waitlist, rather than the active ingredients of the exercise protocol per se. Future studies employing active control conditions such as stretching or health education would strengthen causal inference.

The absence of differential effects between exercise modalities may reflect insufficient statistical power to detect small differences, despite our sample size calculation. However, equivalence testing confirmed that any true difference falls within bounds of negligible practical significance. Additionally, our mediation analyses examined only psychological resilience as a mediator. Future research should investigate multiple parallel mediators including self-efficacy, sleep quality, and neurobiological markers to develop comprehensive models of exercise effects. The predominance of students with mild to moderate symptoms limits conclusions about effectiveness for severe depression or anxiety, populations that may require more intensive or combined interventions (Josefsson et al., 2014). Finally, we did not assess long-term maintenance of exercise behavior beyond the intervention period. Understanding factors that promote sustained physical activity engagement is critical for translating short-term benefits into enduring lifestyle changes. Additionally, while the mediation analysis incorporated a longitudinal design with the mediator (resilience change from T0 to T1) measured prior to the outcome (post-intervention symptoms at T1), full temporal precedence in the strict causal sense remains a limitation. Both the mediator and the outcome were assessed at the same post-intervention wave (T1), meaning that changes in resilience and changes in symptoms may have co-occurred concurrently rather than sequentially. Although bootstrapped confidence intervals and the temporal ordering of constructs strengthen inference, they do not substitute for experimental manipulation of the mediator or a three-wave design with an independent intermediate time point for mediator assessment. Accordingly, the mediation findings should be interpreted as consistent with a resilience-building mechanism rather than as conclusive causal evidence, and future research employing daily diary methods or experience sampling could clarify the temporal dynamics of resilience and symptom change during exercise interventions.

## Conclusion

This randomized controlled trial provides preliminary evidence suggesting that structured exercise interventions, whether team-based or individual, may produce substantial and clinically meaningful improvements in depression, anxiety, and psychological resilience among Chinese college students. Approximately 45% of mental health benefits appear to be mediated through enhanced resilience, highlighting a key psychological mechanism. These findings support the integration of structured physical activity programming into comprehensive university mental health promotion strategies as an evidence-based, accessible, and cost-effective approach. Both team-based sports and individual aerobic exercise appear equally effective, providing implementation flexibility based on institutional resources and student preferences. Future research priorities include: (1) multi-site RCTs with probability sampling across diverse university settings and cultural contexts to establish external validity; (2) longer follow-up periods (6–12 months) to assess the durability of benefits and identify predictors of sustained physical activity engagement; (3) investigation of neurobiological mediators (e.g., BDNF, cortisol) alongside psychological mechanisms; and (4) cost-effectiveness analyses to inform resource allocation decisions for university mental health policy.

## Data Availability

The raw data supporting the conclusions of this article will be made available by the authors, without undue reservation.
